# Knowledge of Human Papillomavirus and Cervical Cancer among Women Attending Gynecology Clinics in Pretoria, South Africa

**DOI:** 10.3390/ijerph19074210

**Published:** 2022-04-01

**Authors:** Teboho Amelia Tiiti, Johannes Bogers, Ramokone Lisbeth Lebelo

**Affiliations:** 1 Department of Virological Pathology, Sefako Makgatho Health Sciences University, Pretoria 0204, South Africa; tebohotiiti@yahoo.co.za; 2 Laboratory of Cell Biology and Histology, Faculty of Medicine and Health Sciences, University of Antwerp, 2610 Antwerpen, Belgium; john-paul.bogers@uantwerpen.be; 3 Algemeen Medisch Laboratorium (AML), Sonic Healthcare, 2020 Antwerpen, Belgium; 4 Department of Anatomical Pathology, Sefako Makgatho Health Sciences University, Pretoria 0204, South Africa; 5 National Health Laboratory Service, Department of Virological Pathology, Sefako Makgatho Health Sciences University, Pretoria 0204, South Africa; 6 South African Vaccination and Immunization Centre, Sefako Makgatho Health Sciences University, Pretoria 0204, South Africa

**Keywords:** HPV, knowledge, cervical cancer, pap smear, risk factors, policy

## Abstract

Background: Cervical cancer is mainly caused by human papillomavirus (HPV). Worldwide, knowledge of HPV and cervical cancer among women is reported to be inadequate. The study aimed to assess the knowledge and awareness of HPV and cervical cancer among women attending gynecology clinics at a tertiary hospital in Pretoria, South Africa. The study also intended to identify socio-demographic factors influencing women’s knowledge about HPV and cervical cancer risk factors. Methods: This was a clinic-based analytic cross-sectional study conducted among women aged 18 years and older. Participants were enrolled in the clinic waiting rooms while waiting to be attended to by the clinician. A self-administered questionnaire to assess knowledge of HPV, cervical cancer, and risk factors for developing cervical cancer was distributed to the participants. Results: A total of 527 women aged ≥18 years and older were randomly enrolled with a 99.8% response rate. Less than half (47.1%) of the participants had been previously screened for cervical cancer using a Papanicolaou (Pap) test. Few (18.8%) women correctly mentioned cervical cancer risk factors. Unemployed women were less likely to have correct knowledge of cervical cancer causes/risk factors (OR: 0.63; 95% CI 0.40–0.97) compared to employed women. Divorced/separated/widowed women were more likely to have good HPV knowledge compared to single participants (OR: 2.74; 95% CI 1.46–5.15). Conclusion: From this study, it is evident that cervical cancer screening is very low, and women lack knowledge of HPV and cervical cancer disease symptoms and its risk factors. There is a need for policies to prioritize providing accurate information to the public to reduce cervical cancer morbidity and mortality.

## 1. Introduction

Cervical cancer is the second most common cancer in South Africa, with 10,702 cases diagnosed annually (estimation for 2020) [1]. Despite successful efforts in reducing cervical cancer, the crude incidence rate per 100,000 South African women is 35.6 [1]. In sub-Saharan regions of Africa, the incidence and mortality of cervical cancer are expected to rise due to the high incidence of human immunodeficiency virus (HIV) infection [2].

In high-income countries (HICs), cervical cytology programs have reduced the incidence and mortality of cervical cancer [3]. However, in high-risk (hr) low- and middle-income countries (LMICs), there are inclining trends in incidence due to the lack and ineffectiveness of screening programs [3]. Moreover, the unavailability of sufficient resources and infrastructure contributes to weakened screening programs [4]. In 2000, the South African National Department of Health (NDoH) launched a national screening program to reduce the incidence of cervical cancer. The policy recommended three free Pap smears to asymptomatic women, within 10-year intervals, starting at age 30. In 2017, the policy was revised, making provision for HIV-positive women, whereby all HIV-positive women are screened upon diagnosis and subsequently, every three years, provided the screening test is negative [5]. In South Africa, two HPV vaccines are available: Cervarix^®^ (GlaxoSmithKline Biologicals, Rixensart, Belgium) and Gardisil^®^ (Merck & Co., Whitehouse Station, NJ, USA). HPV vaccination of girls aged nine to 12 is also recommended in the updated policy [5].

Worldwide, human papillomavirus (HPV) is one of the most common sexually transmitted infections (STIs) in both men and women [6]. HPVs are stratified according to their oncogenic potential, hence high-risk (hr) HPVs include the genotypes that cause malignancy, and low-risk (lr) HPVs include those genotypes that cause common, flat plane and anogenital warts [7]. Infection with hr-HPV is implicated in nearly all cervical cancers [8]. However, infection with HPV alone may not be a sufficient cause of cervical cancer [9]. HPV infection with the presence of various cofactors such as the use of oral contraceptives (OCs), tobacco smoking, diet, cervical trauma, and coinfection with HIV and other sexually transmitted agents may influence the risk of progression from cervical HPV infection to cervical cancer [9]. Cervicovaginal microbiota [10]; obesity, although the association with an increased risk of cervical cancer is weak [11]; and immunosuppression [12] are amongst cervical cancer factors. Furthermore, infection with HPV in pregnant women is associated with the risk of spontaneous abortion [13].

Regarding knowledge of HPV, many women fail to understand the causal relationship between HPV infection and cervical cancer [14]. Furthermore, in African societies, women often cite incorrect risk factors for cervical cancer such as vaginal douching and insertion of substances in the vagina [15]. Moreover, an important risk factor for increasing cervical cancer incidence is the nonattendance of participants in screening programs [16]. Inconvenient appointment times, education and knowledge, fear, and embarrassment are some of the factors that impede effective screening [17]. Several studies indicate that there is a significant lack of HPV knowledge in sub-Saharan Africa (SSA) [18,19,20]. In African countries, low levels of education contribute to the high incidence and mortality of the disease [21]. South African studies among rural and women attending tertiary institutions have reported low cervical cancer knowledge [22,23].

As a plan to eliminate cervical cancer, all countries must reach and maintain an incidence rate of below 4 per 100,000 women. Currently, South Africa has an incidence rate of 35.6 per 100,000 women [1]. To achieve the World Health Organization’s (WHO) goals to eliminate the disease, three steps are outlined: (i) prevention through vaccination, (ii) screening and treatment of precancerous lesions, and (iii) treatment of precancerous lesions treatment and palliative care for invasive cervical cancer. For the goals to be achieved, policies need to be developed to address these goals. The target population must have accurate information and knowledge to be able to make decisions that would prevent the disease and take into consideration the available measures that are in place to treat and manage the cervical disease. These measures would subsequently reduce the burden of cervical cancer. It is therefore important that countries develop policies and educational programs to inform and educate the population. Thus, studies should be conducted to evaluate the knowledge levels of different populations to develop a program that would be effective. This study aimed to evaluate women’s knowledge of HPV and cervical cancer. The study also intended to identify socio-demographic factors influencing women’s knowledge about HPV and cervical cancer risk factors.

## 2. Materials and Methods

This was an analytic cross-sectional study conducted between March 2016 and September 2018, targeting women aged 18 years and older attending gynecology clinics at a tertiary hospital in Pretoria, South Africa. The cross-sectional study has been described in detail previously [24]. The Sefako Makgatho Health Sciences Research and Ethics Committee approved the study (SMUREC/P/75/2016: IR). Participants attended a Gynecological Oncology clinic for review of their Pap smear after treatment with large loop excision of the transformation zone (LLETZ); colposcopy examination after a positive Pap smear; cervical cancer screening using a Pap smear; and termination of pregnancy, family planning, and other gynecology-related services at the TOP (termination of pregnancy) clinic. Women who had undergone a complete hysterectomy or were going through their monthly menstrual cycle were excluded.

This study is part of an overarching study that collected two samples: a clinician sample using the Cervex Brush^®^ Combi (Rovers^®^ Medical Devices B.V., Lekstraat, The Netherlands) and self-collected samples using the SelfCerv, applicator tampon (Ilex Medical Ltd., Johannesburg, South Africa). The overarching goal of the study was to evaluate the performance of self-collected samples for human papillomavirus (HPV) detection, and further investigate women’s preferences and experiences of self-sampling; evaluate HPV prevalence; and assess knowledge of HPV and cervical cancer among gynecology clinic attendees. The purpose of this substudy was to assess knowledge of HPV and cervical cancer risk factors among women seeking gynecology services. A self-administered questionnaire was initially developed in English, then translated into the local language, and then translated back to English for quality assurance. The questionnaire included a section on socio-demographic characteristics such as age and race, a section on screening history, and knowledge of HPV and cervical cancer. To determine participants’ knowledge of cervical cancer, questions on causes/risk factors of developing cervical cancer and symptoms were included. The question on causes/risk factors of developing cervical cancer was open-ended and participants were scored “1” for each correct risk factor and “0” for the incorrect risk factor mentioned, respectively. Knowledge of HPV was assessed using the following questions: “What is human papillomavirus?”, “Do you think HPV testing shows that you have cervical cancer?”, and “It is possible for HPV to cause cancer?”. According to the number of correct answers, the sum of scores of the 3 items was categorized as follows: 0 = “no knowledge”, 1 = “fair knowledge”, 2 = “good knowledge” and 3 = “very good knowledge”. HPV knowledge was categorized as accurate versus inaccurate. “Yes” was considered accurate and “No” or “Don’t know” inaccurate for the question: “It is possible for human papillomavirus to cause cancer?”. “No” was classified as accurate, and “Yes” or “Don’t know” as inaccurate for the question: “Do you think HPV testing shows that you have cervical cancer?”.

The questionnaire was distributed to participants after the study had been explained. Participants were asked to fill in the section of the questionnaire on demographics and knowledge of HPV and cervical cancer as they were waiting to self-collect and be examined by a clinician. The section on self-sampling experiences and preferences was filled in after participants had both self-collected and provided a clinician-collected sample.

### Data Analysis

Data were analyzed using SAS version 9.4 for Windows. Descriptive statistics were used to summarize all variables of interest in the study population. The association between Pap smear (cervical cancer screening) and the age group was tested using the chi-square test. Odds ratios (unadjusted and adjusted) and 95% CIs for potential determinants associated with study outcomes were estimated using the logistic regression model. Variables significant at a significant cut-off of 0.2 in the bivariate regression analyses were selected for inclusion into the final multivariable model. An adjusted *p*-value of <0.05 was deemed statistically significant.

## 3. Results

### 3.1. Demographic Characteristics of Participants

In this study, data of 527 women were collected with a 99.8% response rate. Therefore, 526 participants were included in the analysis (Table 1). Most (32.7%) of the participants were between the ages of 30 to 39 years, and the age group ≥ 30 years that are eligible for cervical cancer screening in South Africa comprised 71.1% of the study participants. The mean age was 36.8 years. The majority (99.6%) of the participants were black, 67.3% were single, and more than half (57.4%) had either one or two children. Less than half (45.1%) of the participants were employed and 85.6% of the participants reported residing in semi-urban areas.

### 3.2. Knowledge of HPV among the Study Participants

Generally, knowledge of HPV infection was insufficient (Table 2). In this study, it was found that 138 respondents (26.2%) know what HPV is; and 192 respondents (36.5%) answered “Yes” to the question whether HPV testing shows that one has cervical cancer. Less than half (45.6%) of the respondents knew that HPV is a causative agent of cervical cancer, and 42.6% did not know whether HPV is a causative agent of cervical cancer or not. About 60% of the participants had knowledge of HPV. Overall, 40.3% of participants had no knowledge of HPV, with most (32.3%) of the participants having fair knowledge and only 6.3% having very good knowledge.

### 3.3. Knowledge of HPV According to Pap Screening

Women who had been previously screened by a Pap smear were more likely to have very good knowledge of HPV (60.6%) compared to those who had not been screened (39.4%). Those who had not previously been screened for cervical cancer were more likely to have poor knowledge (57.6%) compared to the previously screened group (42.4%). There was a significant association between previous Pap screening and HPV knowledge (*p* = 0.010). As shown in Table 3, women seeking family planning services (36.7%) and termination of pregnancy (39.1%) were more likely to have a fair knowledge of HPV. The results further show that women undergoing review with a Pap smear following treatment were more likely to have good (31.6%) and very good (12.3%) knowledge of HPV compared to the other groups. Knowledge of HPV was statistically associated with the reason for clinic visits (*p* = 0.004).

Although there was no statistical difference between knowledge of cervical cancer risk factors with Pap smear status (*p* = 0.063), women who had not previously had a Pap smear (22.2%) were more likely not to know the correct risk factors of cervical cancer. Women who were attending the clinic for review with Pap smear after treatment (26.3%) and routine Pap smear (24.6%) were more likely to know the correct risk factors (*p* = 0008).

### 3.4. Screening History and Knowledge about Cervical Cancer Risk Factors

Less than half (47.1%) of the participants reported having been screened for cervical cancer previously, and of the screened participants, most (43.0%) had been screened ≤5 years ago. There was a significant association between previous screening with a Pap smear and age (*p* < 0.0001; Cramer’s V = 0.38). Regarding signs and symptoms of cervical cancer, participants were asked whether women with vaginal discharge and/or bleeding need to be screened for cervical cancer; the majority (58.8%) of the participants answered “No” and 13.7% answered, “Don’t know”. Furthermore, the participants were asked about the causes of cervical cancer/risk factors leading to cervical cancer development (Table 4). A low proportion (18.8%) of the participants mentioned correct risk factors. Only 16.0% of the participants mentioned one correct risk factor, 2.7% mentioned two, and only 0.2% mentioned three correct risk factors. STIs (8.6%), HPV (4.6%), and multiple sexual partners (4.0%) were the most frequent risk factors mentioned by respondents. Only 0.2% of the participants mentioned HIV, multiple births, and immunosuppression as causes of cervical cancer/risk factors leading to cervical cancer development, respectively. Few (13.1%) participants reported being screened for HPV previously and of the screened participants, 11.6% were screened ≤ 5 years ago. Thirty-seven (7.0%) of the previously screened participants reported that the test had been suggested by a healthcare worker. Most (68.8%) of the participants who had not been tested for HPV expressed the need to be tested for HPV. Seventy (13.3%) participants reported being afraid that HPV testing may be suggestive of cervical cancer, while 36.3% reported being at risk of HPV acquisition.

### 3.5. Association between Knowledge of HPV and Socio-Demographic Characteristics

There was no significant association between age, place of residence, and the number of children a woman has and HPV knowledge (Table S1). In the univariate analysis, there was a significant association between knowledge of HPV and marital status. Divorced/widowed/separated participants were more likely to have good knowledge of HPV compared to single participants (OR 2.74; 95% CI 1.46–5.15; *p* = 0.0017). Unemployed participants were less likely to have good knowledge compared to employed participants (OR 0.68; 95% CI 0.46–0.995; *p* = 0.047). Alternatively, in a full multivariate analysis with all five socio-demographic variables, only marital status was significant, with both married and divorced/widowed/separated women being more likely to have good knowledge compared to single participants when controlling for other variables in the model.

### 3.6. Association of Knowledge on Causes of Cervical Cancer and Socio-Demographic Characteristics

In the univariate analysis, unemployed participants were less likely to have correct knowledge of cervical cancer risk factors compared to employed participants (OR 0.63; 95% CI 0.40–0.97). Only employment status remained significantly associated with the knowledge of cervical cancer risk factors in the multivariable analysis. When fitted in a full model, employment status remained significant. Age, marital status, place of residence, and the number of children a woman has were not significantly associated with knowledge of cervical cancer risk factors (Table S2).

## 4. Discussion

The current study assessed knowledge of HPV infection, cervical cancer, and its risk factors among women attending gynecology clinics in Pretoria, South Africa. The study also intended to identify socio-demographic factors influencing women’s knowledge of HPV and cervical cancer risk factors. Key findings from this study indicate that gynecology clinic attendees (i) are unfamiliar with HPV infection, cervical cancer risk factors, and its symptoms, and (ii) half of the participants reported not being screened for cervical cancer in their lifetime.

The study highlighted that the majority (73.8%) of participants in the current study did not know what HPV is. The lack of knowledge of HPV in this group of women may place them at an increased risk of contracting HPV infection. Similarly, previous studies conducted in SSA reported low knowledge of HPV infection in LMICs [20,25,26]. Most of the participants in the current study (72.6%) lacked adequate knowledge and understanding of HPV infection. In South Africa, a previous study that evaluated knowledge of HPV and HPV infection reported women having better knowledge (62.5%) compared to the current study [22]. However, the findings reported by Mofolo et al. are expected as the study population included university students who may have had exposure to health education and programs at a higher institution [22]. Over half of the participants (54.4%) could not link HPV infection with the development of cervical cancer. Previous studies in Nigeria and South Africa among women attending healthcare clinics showed that 91.5% and 97.8% of the participants, respectively, lacked knowledge about HPV topics including HPV infection, HPV vaccine, and transmission, showing a lower level of knowledge compared to the current study [19,20]. Mingo et al. reported that only 3 participants among 289 women attending public health clinics (1%) linked HPV to cervical cancer [14].

Women who had previously accessed Pap screening services were more likely to have better knowledge of HPV compared to those who had never been screened prior to the study. Information may have been provided during screening, and thus, might explain why these women had better knowledge. There was a link between not being screened and a lack of information about cervical cancer risk factors. Furthermore, women who underwent a Pap smear following treatment with LLETZ were more likely to have better knowledge of HPV and cervical cancer risk factors. These women had previously been diagnosed with abnormal lesions; hence, it is not surprising that these group of women seemed to have better knowledge, as they may have been exposed to accurate information during the period of having a Pap smear test. Furthermore, when investigating determinants of knowledge of HPV, having good HPV knowledge was statistically associated with marital status.

A regular Pap smear can prevent the most invasive cancers of the cervix [27]. Asymptomatic women over the age of 30 years are eligible to undergo three free Pap smear tests in the country’s public health sector, and all HIV-positive women are screened upon diagnosis and subsequently, every three years, provided the screening test is negative [5]. However, data from the current study show that 52.9% of the participants had not been screened and 41% of unscreened women were over the age of 30 years. There was a significant association between previous screening with Pap smear and age. It was not surprising that the proportion of women who had been screened was higher in the age group of 40 years and older compared to those aged 30 years and less; as previously mentioned, the Cervical Cancer Prevention and Control Policy in the country recommends screening for women aged 30 years and older. Our findings are consistent with previous studies that reported low uptake of cervical screening in SSA [28,29,30,31]. The high number of unscreened participants is concerning and hence requires intervention, appropriate measures, and policies in place given the high rates of cervical cancer in SSA. Regarding symptoms of cervical cancer, only 27.6% of the participants associated vaginal discharge and/or vaginal bleeding with cervical cancer. This shows that most of the participants may not be able to differentiate between normal and abnormal bleeding or discharge. This might be due to the lack of health education programs regarding cervical cancer. Contrary to the current study, a study conducted in Ethiopia reported 43.6% of the participants mentioning either vaginal bleeding or foul-smelling vaginal discharge as symptoms of cervical cancer [32].

Few (18.8%) participants were able to mention at least one or more correct risk factors for developing cervical cancer. Any other two risk factors were reported by 2.7% and any three factors were reported by 0.2%. Contrarily, Hoque et al. reported higher figures (21% for any other two risk factors and 16% for any three risk factors) [33]. Our findings show a very low level of knowledge of cervical cancer risk factors, despite the existence of the South African National Guidelines on Cervical Cancer Screening. This suggests that information about cervical cancer and its causes needs to be available to women, particularly those at higher risk of acquiring HPV infection and developing cervical cancer. Previous studies from various countries have reported better awareness of cervical cancer risk factors compared to our study: Ethiopia (31% and 41.9%), and South Africa (64.0%) [27,32,33]. However, almost similar results were reported in the Democratic Republic of Congo (19.3%) [34]. The discrepancy might be explained by the differences in the background of study participants, such as educational level, occupation, and other factors that may influence access to health education. In the current study, participants commonly mentioned risk factors such as HPV infection, STIs, multiple sexual partners, and unprotected sexual intercourse. Risk factors such as HIV, multiple births (multiparity), early sexual debut, immunosuppression, and heredity were less frequently mentioned. Knowledge of the risk factors and symptoms of cervical cancer might make a substantial difference in the population and consequently reduce the burden of cervical cancer, particularly in populations most affected by cervical cancer. It is surprising that 45.6% of participants correctly answered that HPV can cause cancer, while only 4.6% of the participants mentioned HPV as a risk factor for cervical cancer. This might imply that the questions were not presented clearly to the participants or at the level of their education. However, since the education level of the participants was not established, this cannot be verified.

HPV testing is relatively new in South Africa, particularly in the public sector, and the rollout of HPV testing has recently been proposed [5]. Overall, 86.9% of the participants had not been screened for HPV previously, which is not surprising given the status of HPV testing in the public sector. However, the current study’s findings show that there are women who attend both public and private health facilities as other participants reported having been tested for HPV previously, which is not available at public health facilities in the country as yet. Most of the participants who were tested for HPV stated that the test was suggested by a healthcare worker, which indicates opportunistic testing probably when patients were consulting a healthcare worker at a private hospital or practice. Most (36.3%) participants may have been aware of the risk of having acquired HPV infection, while other participants (40.5%) did not know whether they were at risk of having an infection or not. It is therefore important to increase awareness of HPV through social media, community outreach, health talks, posters, and information leaflets at hospitals and clinics, and more sensitization through healthcare workers. Seventy of the participants (13.3%) reported being afraid that HPV testing will make people think that they have cervical cancer; this indicates a poor understanding of HPV and cervical cancer topics. Although HPV testing has not been widely introduced in the public sector, secondary prevention by strengthening screening methods, including the introduction of HPV testing in the public sector, is recommended by the NDoH. Hence, this shows that there is a need for the dissemination of education and proper information in the community for people to accept and respond positively to HPV screening. Older women (general population) were most likely to have good knowledge of cervical cancer and its risk factors, which is consistent with previous studies [30,35]. Employment status remained significantly associated with knowledge of cervical cancer risk factors, which has been previously reported [30].

Our study was clinic-based and aimed at assessing the knowledge of HPV and cervical cancer among gynecology clinic attendees. Hence, these results cannot be generalized to the general population. Educational level was not included in the questionnaire; thus, we could not determine whether HPV and cervical cancer knowledge were influenced by educational level. Since the participants were women attending gynecologic clinics and enrolled for HPV testing, and hence, they were informed about HPV testing and its significance, this is recognized as a major bias in the study. The HPV vaccination status of participants was not included.

## 5. Conclusions

In conclusion, although cervical cancer screening is the most effective approach to cervical cancer control and this is addressed in the Cervical Cancer and Prevention Control Policy in South Africa, the screening uptake in the study was low. Furthermore, the study found that the knowledge of HPV and cervical cancer among women attending gynecology clinics was low. Government policies must prioritize providing accurate information and awareness to the public, and should recommend resources towards issues mostly associated with lack of knowledge of HPV and cervical cancer. Eliminating knowledge-related barriers is important to achieve the goal of reducing cervical cancer incidence and mortality rates.

## Figures and Tables

**Table 1:** Socio-demographic characteristics of the study participants.

Variables	n	%
**Age (years)**		
<30	151	28.9
30–39	171	32.7
40–49	121	23.1
50–59	61	11.7
≥60	19	3.6
Eligible for screening (≥30)	372	71.1
Number missing	3	
**Race**		
Black African	524	99.6
Other	2	0.4
**Marital Status**		
Single	354	67.3
Married	126	24.0
Divorced/Widowed/Separated	46	8.8
**Employment Status**		
Employed	237	45.1
Unemployed	289	54.9
**Place of residence**		
Rural	12	2.3
Semi-Urban	447	85.6
Urban	63	12.1
Number missing	4	
**Number of Children**		
None	92	17.5
1–2	302	57.4
3–4	118	22.4
≥5	14	2.7

**Table 2:** Knowledge of HPV.

Question	n	%
**What is the human papillomavirus?**		
Correct	138	26.2
Incorrect	388	73.8
**Do you think HPV testing shows that you have cervical cancer?**		
Yes	192	36.5
No	113	21.5
Don’t know	221	42.0
**It is possible for human papillomavirus to cause cancer?**		
Yes	240	45.6
No	62	11.8
Don’t know	224	42.6
**HPV knowledge score**		
Very good knowledge	33	6.3
Good knowledge	111	21.1
Fair knowledge	170	32.3
No Knowledge	212	40.3

**Table 3:** Knowledge of HPV and cervical cancer risk factors by Pap screening and reason for the clinic visit.

	n	Knowledge of HPV				*p*-Value
		No Knowledge	Fair Knowledge	Good Knowledge	Very Good Knowledge	
**Pap screening**						0.010
Yes	248	90 (42.4)	73 (42.9)	65 (58.6)	20 (60.6)	
No	278	122 (57.6)	97 (57.1)	46 (41.4)	13 (39.4)	
**Reason for the clinic visit**						
Colposcopy	33	17 (51.5)	8 (24.2)	8 (24.2)	0 (0)	0.004
Family planning	30	9 (30)	11 (36.7)	7 (23.3)	3 (10.0)	
LLETZ	87	46 (52.9)	25 (28.7)	13 (14.9)	3 (3.5)	
Review with Pap smear	57	18 (31.6)	14 (24.6)	18 (31.6)	7 (12.3)	
Routine Pap smear	232	82 (35.3)	78 (33.6)	52 (22.4)	20 (8.6)	
Termination of pregnancy	87	40 (46.0)	34 (39.1)	13 (14.9)	0 (0.0)	
		**Knowledge of Cervical Cancer Risk Factors**				
**Pap screening**		Incorrect	Correct			0.063
Yes	248	193 (77.8)	55 (22.2)			
No	278	234 (84.2)	44 (15.8)			
**Reason for the clinic visit**						0.008
Colposcopy	33	29 (87.9)	4 (12.1)			
Family planning	30	27 (90.0)	3 (10.0)			
LLETZ	87	77 (88.5)	10 (11.5)			
Review with Pap smear	57	42 (73.7)	15 (26.3)			
Routine Pap smear	232	175 (75.4)	57 (24.6)			
Termination of pregnancy	87	77 (88.5)	10 (11.5)			

**Table 4:** Screening history and knowledge about cervical cancer risk factors.

Question	n	%
**Have you been screened for cervical cancer before using a Pap smear?**		
Yes	248	47.1
No	278	52.9
**Participants screened for cervical cancer categorized by age** (*p* < 0.0001; Cramer’s V = 0.38)		
<30 years	30	19.9
30–39 years	84	49.1
40–49 years	80	66.1
50–59 years	42	68.9
≥60 years	12	63.2
**If yes, when was the last time you had a Pap smear?**		
1–5 years	226	43.0
6–10 years	18	3.4
11–15 years	2	0.4
16–20 years	1	0.2
21–25 years	1	0.2
**Do you think women with vaginal discharge and/or bleeding need to be screened for cervical cancer?**		
Yes	145	27.6
No	309	58.7
Don’t know	72	13.7
**What is your knowledge of the cause of cervical cancer /risk factors for cervical cancer?**		
Knowledgeable (correct)	99	18.8
Not Knowledgeable (incorrect)	427	81.2
**Number of correct risk factors mentioned by the participants**		
One	84	16.0
Two	14	2.7
Three	1	0.2
**Risk factors for cervical cancer mentioned by the participants**		
HPV	24	4.6
Co-infection with HIV (human immunodeficiency virus)	1	0.2
STIs	45	8.6
Unprotected sexual intercourse	11	2.1
Multiple Sexual Partners	21	4.0
Multiple births	1	1.0
Early sexual debut	5	1.0
Immunosuppression	1	0.2
Family history or heredity	6	1.1
**Have you been tested for HPV before?**		
Yes	69	13.1
No	457	86.9
**How long ago were you tested?**		
1–5 years	61	11.6
6–10 years	8	1.5
**Who suggested for you to be tested for HPV?**		
Healthcare worker	37	7.0
Yourself	28	5.3
Family/friend/colleague	4	0.8
**Do you feel that you need to be tested for HPV?**		
Yes	362	68.8
No	25	4.8
Don’t know	70	13.3
**Are you afraid that HPV testing will make people think you have cervical cancer?**		
Yes	70	13.3
No	456	86.7
**Do you think that you are at risk of having an HPV infection?**		
Yes	191	36.3
No	122	23.2
Don’t know	213	40.5

## Data Availability

The datasets generated during and/or analyzed during the current study are available from the corresponding author on reasonable request.

## Supplementary Materials

The following supporting information can be downloaded at: https://www.mdpi.com/article/10.3390/ijerph19074210/s1, Table S1: Association of knowledge of HPV and socio-demographic characteristics; Table S2: Association between knowledge of causes of cervical cancer and socio-demographic variables.
