# Supplementary material for: Knowledge of Human Papillomavirus and Cervical Cancer among Women Attending Gynecology Clinics in Pretoria, South Africa
Source: Int J Environ Res Public Health. 2022 Apr 1;19(7):4210. doi: 10.3390/ijerph19074210 (PMC8998542; doi:10.3390/ijerph19074210)
Supplement: Supplementary file 1 [file ijerph-19-04210-s001.zip › ijerph-1569036-supplementary.pdf]

Table S1. Association of knowledge of HPV and socio-demographic characteristics

	Overall	Knowledge: Fair/None	Knowledge: (Very) Good	Univariate Models			Multivariable Model (Reduced)			Multivariable Model (Full)		
**Variable**	**N (%)**	**n (%)**	**n (%)**	***p*-value**	**OR (Very) Good Knowledge**	**95% CI OR**	**p-value**	**OR (Very) Good Knowledge**	**95% CI OR**	***p*-value**	**OR (Very) Good Knowledge**	**95% CI OR**
**Age (years)**	**526**	**382 (72.6)**	**144 (27.4)**									
<30	151 (28.9)	111 (73.5)	40 (26.5)		1.00	reference					1.00	reference
30-39	171 (32.7)	121 (70.8)	50 (29.2)	0.58	1.15	0.70 1.87				0.72	0.90	0.52 1.57
40-49	121 (23.1)	86 (71.1)	35 (28.9)	0.66	1.13	0.66 1.93				0.34	0.73	0.38 1.40
50-59	61 (11.7)	48 (78.7)	13 (21.3)	0.43	0.75	0.37 1.53				0.054	0.43	0.18 1.02
60-68	19 (3.6)	13 (68.4)	6 (31.6)	0.64	1.28	0.46 3.60				0.50	0.67	0.21 2.12
not specified	3											
**Marital status**												
Single	354 (67.3)	271 (76.6)	83 (23.4)		1.00	reference		1.00	reference		1.00	reference
Married	126 (24.0)	86 (68.3)	40 (31.7)	0.068	1.52	0.97 2.38	0.068	1.52	0.97 2.38	0.031	1.74	1.05 2.88
Divorced/Widowed/ Separated	46 (8.8)	25 (54.3)	21 (45.7)	0.0017	2.74	1.46 5.15	0.0017	2.74	1.46 5.15	0.0022	3.07	1.50 6.28
**Employment status**												
Employed	237 (45.1)	162 (68.4)	75 (31.6)		1.00	reference					1.00	reference
Unemployed	289 (54.9)	220 (76.1)	69 (23.9)	0.047	0.68	0.46 0.995				0.094	0.71	0.47 1.06
**Place of residence**												

Semi-urban	447 (85.6)	327 (73.2)	120 (26.8)		1.00	reference		1.00	reference
Semi-rural	63 (12.1)	42 (66.7)	21 (33.3)	0.28	1.36	0.78 2.40	0.30	1.36	0.76 2.43
Rural	12 (2.3)	9 (75.0)	3 (25.0)	0.89	0.91	0.24 3.41	0.93	0.94	0.24 3.66
not specified	4								
Number of children									
No child	92 (17.5)	66 (71.7)	26 (28.3)		1.00	reference		1.00	reference
1 or 2 children	302 (57.4)	220 (72.58)	82 (27.2)	0.83	0.95	0.56 1.59	0.67	0.88	0.50 1.56
3 or 4 children	118 (22.4)	87 (73.7)	31 (26.3)	0.75	0.91	0.49 1.67	0.73	0.88	0.44 1.78
5 and more	14 (2.7)	9 (64.3)	5 (35.7)	0.57	1.41	0.43 4.61	0.71	1.29	0.34 4.96

Table S2. Association between knowledge of causes of cervical cancer and socio-demographic variables.

	Overall	Knowledge: CORRECT	Knowledge: Incorrect	Univariate Models			Multivariable Model (reduced)			Multivariable Model (full)		
**Variable**	**n**	**N**	**n**	***p*-value**	**OR Correct Knowledge**	**95% CI OR**	***p*-value**	**OR Correct Knowledge**	**95% CI OR**	***p*-value**	**OR Correct Knowledge**	**95% CI OR**
**Age (years)**	**526**	**99 (18.8)**	**427 (81.2)**									
<30	151 (28.9)	32 (21.2)	119 (78.8)		1.00	reference					1.00	reference
30-39	171 (32.7)	26 (15.2)	145 (84.8)	0.16	0.67	0.38 1.18				0.12	0.61	0.32 1.15
40-49	121 (23.1)	28 (23.1)	93 (76.9)	0.70	1.12	0.63 1.99				0.91	0.96	0.48 1.94
50-59	61 (11.7)	8 (13.1)	53 (86.9)	0.18	0.56	0.24 1.30				0.11	0.45	0.17 1.20
60-68	19 (3.6)	5 (26.3)	14 (73.7)	0.61	1.33	0.45 3.96				0.89	1.09	0.32 3.69
not specified	3											
**Marital status**												
Single	354 (67.3)	60 (16.9)	294 (83.1)		1.00	reference					1.00	reference
Married	126 (24.0)	27 (21.4)	99 (78.6)	0.26	1.34	0.80 2.22				0.16	1.51	0.85 2.69
Divorced/Widowed/Separated	46 (8.8)	12 (26.1)	34 (73.9)	0.13	1.73	0.85 3.53				0.18	1.74	0.77 3.91
**Employment status**												
Employed	237 (45.1)	54 (22.8)	183 (77.2)		1.00	reference		1.00	reference		1.00	reference
Unemployed	289 (54.9)	45 (15.6)	244 (84.4)	0.036	0.63	0.40 0.97	0.036	0.63	0.40 0.97	0.049	0.63	0.40 0.997
**Place of residence**												
Semi-urban	447 (85.6)	87 (19.5)	360 (80.5)		1.00	reference					1.00	reference

Semi-rural	63 (12.1)	10 (15.9)	53 (84.1)	0.50	0.78	0.381.60	0.51	0.78	0.38	1.62
Rural	12 (2.3)	2 (16.7)	10 (83.3)	0.81	0.83	0.183.85	0.83	0.85	0.17	4.11
not specified	4									
**Number of children**										
No child	92 (17.5)	21 (22.8)	71 (77.2)		1.00	reference		1.00	reference	
1 or 2 children	302 (57.4)	56 (18.5)	246 (81.5)	0.36	0.77	0.441.36	0.36	0.75	0.41	1.39
3 or 4 children	118 (22.4)	19 (16.1)	99 (83.9)	0.22	0.65	0.331.30	0.25	0.63	0.29	1.39
5 and more	14 (2.7)	3 (21.4)	11 (78.6)	0.91	0.92	0.243.62	0.97	0.98	0.21	4.49
